# Patient and Caregiver Education to Support Self‐Efficacy and Self‐Management During Immunotherapy—An Integrative Review

**DOI:** 10.1002/pon.70100

**Published:** 2025-02-26

**Authors:** Camilla S. Rothausen, Karin B. Dieperink, Christina H. Ruhlmann, Helle Pappot, Lærke K. Tolstrup

**Affiliations:** ^1^ Department of Clinical Research University of Southern Denmark Odense Denmark; ^2^ Department of Oncology Odense University Hospital Odense Denmark; ^3^ FaCe ‐ Family Focused Healthcare Research Center University of Southern Denmark Odense Denmark; ^4^ Immunotox Research Center of Southern Denmark (ICiTox) Odense University Hospital Odense Denmark; ^5^ Department of Oncology Rigshospitalet Copenhagen Denmark; ^6^ Department of Clinical Medicine University of Copenhagen Copenhagen Denmark

**Keywords:** cancer, caregiver education, immune checkpoint inhibitors, immunotherapy, oncology, patient education, self‐efficacy, self‐management

## Abstract

**Background:**

Immune checkpoint inhibitors (ICI) have improved cancer treatment, but this treatment can lead to immune‐related adverse events (irAEs). Effective patient and caregiver education is essential to better management of irAEs and improved treatment outcomes.

**Objective:**

This integrative review aimed to elucidate how patient education on ICI efficacy and toxicity management affects patients with cancer and their family caregivers' self‐efficacy and self‐management when dealing with irAEs.

**Methods:**

An integrative review was conducted. EMBASE, MEDLINE, CINAHL, PsycINFO, and Scopus were searched for original research articles. Studies on educational interventions related to ICI and how it affects self‐efficacy and self‐management of irAEs were included. Data were analyzed using thematic analysis.

**Results:**

Of 4182 references screened; seven studies were included. Three themes emerged: (a) Feasibility of various strategies in patient education, (b) The effect of patient education on self‐efficacy, and (c) Determinants to improve self‐management of irAEs.

**Conclusion:**

While traditional patient education methods (oral and/or written information) remain valuable, integrating digital technologies is promising to enhance understanding of ICI. Patient education, especially when combined with follow‐up, can improve health‐related quality of life and self‐efficacy. However, health literacy plays a critical role in treatment and management of irAEs, emphasizing the need for personalized education approaches.

**Trial Registration:**

The protocol is registered with PROSPERO (No. CRD42024511513)

**Abbreviations**
ePRO: electronic Patient Reported Outcomes
ICI: immune checkpoint inhibitors
irAEs: immune‐related adverse events
mHealth: mobile health applications
VR: virtual reality

## 1 Background

The first immune checkpoint inhibitor (ICI) therapy, often referred to as immunotherapy, was approved for the treatment of metastatic melanoma in 2011 [1]. ICIs block negative signaling pathways in the immune system enabling the T‐cells to kill cancer cells [2]. The side effects triggered by ICIs differ significantly from the side effect profiles associated with classic cytostatic cancer treatments. This distinction is inherent to the mechanism of action of ICIs. The interference with the immune system, blocking essential immune checkpoints (e.g., cytotoxic T‐lymphocyte‐associated protein 4, programmed cell death protein 1, or programmed death‐ligand 1), can induce an immune response to the organs of the body, mimicking autoimmune diseases [2]. The side effects caused by ICIs are referred to as immune‐related adverse events (irAEs), and their occurrence is unpredictable, but seen most during the first 3–4 months after ICI initiation [3]. A variety of cancer diseases are now treated with ICI as monotherapy, as ICI combination therapy, or in combination with other antineoplastic treatments [4].

The risk of severe irAEs varies between 15% and 59% depending on the type of ICI and whether it is given as mono‐ or combination therapy [5]. IrAEs are potentially life‐threatening, can necessitate admission to the emergency department or hospitalizations, influence treatment duration [6], and affect the quality of life for patients and caregivers [7]. Patient and caregiver education may ensure identification and prevention of irAEs at an early stage, preventing them from becoming severe [8]. Patient education is the process of influencing patient behavior and producing the changes in knowledge, attitudes and skills necessary to maintain or improve health [9]. In this review, we define patient education on ICI as the process of providing the patient and the appointed family caregivers with the necessary knowledge about ICI, its efficacy, irAEs, and the management of irAEs.

Being diagnosed with cancer is highly distressing for patients and family caregivers [10]. The amount of information correctly recalled by patients about the disease and treatment is strikingly small [11] impacting the management of irAEs [12]. Furthermore, the level of health literacy among patients can influence their ability to understand and manage treatment, impacting their safety throughout the cancer care pathway [13]. Health literacy is the ability to obtain, process and understand medical information [14]. Family caregivers can help patients recall the information received during consultations and discuss key aspects of cancer treatment with them [15], serving an important role as co‐receiver of information. Moreover, studies show that the level of self‐efficacy aligns with symptom occurrence, and high self‐efficacy among patients with cancer is associated with low symptom occurrence and symptom distress [16, 17]. Self‐efficacy is the conviction that one can successfully execute the behavior required to produce certain outcomes [18]. Self‐efficacy empowers individuals to cope or self‐manage with all that a chronic or life‐threatening condition, such as cancer, entails [18]. Thus, high levels of self‐efficacy may lead to better self‐management [19], including more attention to irAEs. Self‐management in this context is defined as the ability to manage the disease and treatment effects and psychosocial changes arising as a result of illness [20].

Previous reviews examined different ways to provide information to patients with cancer. They focused on pre‐consultation interventions [21], the use of mobile health devices and applications (mHealth) [22], and the effects of virtual reality [23] as a patient education tool. To the best of our knowledge, an integrative review regarding education on ICI for patients with cancer including their family caregivers has not been conducted. Furthermore, we identified no previous review regarding how education on ICI affects patients' and family caregivers' self‐efficacy and self‐management of irAEs.

### 1.1 Objective

This integrative review aimed to elucidate the existing knowledge of education of patients with cancer receiving ICI therapy and their family caregivers, focusing on patients' and caregivers' self‐efficacy and self‐management of irAEs. The following research question guided the review:

- **Research Question:** How does patient education on ICI efficacy and toxicity management affect patients with cancer and their family caregivers' self‐efficacy and self‐management when dealing with immune‐related adverse events?

## 2 Methods

### 2.1 Design

This integrative review followed the methodology of Whittemore and Knafl [24]. The Preferred Reporting Items for Systematic Reviews and Meta‐Analysis (PRISMA) guided the reporting of the review, increasing the transparency of the review. The review was prospectively reported to the International Prospective Register of Systematic Reviews (PROSPERO) ref number: CRD42024511513.

### 2.2 Eligibility Criteria

*Inclusion criteria:* Studies addressing how patient education on ICI affects adult patients ≥ 18 years of age with various cancer types and their adult family caregivers ≥ 18 years of age management of irAEs were included. The patients should be treated with ICI as first or second‐line treatment in an oncological (inpatient and/or outpatient) setting. In this study, we defined family according to Wright and Leahey: “*the family is who they say they are*” [25]. Family caregivers could be adult relatives, close friends, neighbors, and the like, who help with self‐care tasks, provision of emotional and social support, health and medical care, advocacy and care coordination, and surrogacy [26]. The review included original research. In accordance with the methodology of integrative reviews, gray literature in the form of conference abstracts from original research is included. There was no restriction to study type (qualitative/quantitative). No geographical limiters were applied.

*Exclusion criteria:* Studies conducted outside of an oncological setting; the study population consisted of pediatric patients or children as family caregivers; or patients were treated with ICI as third or subsequent line treatment. Studies in other language than English, Danish, Norwegian, or Swedish were excluded thereby increasing the feasibility of the review.

### 2.3 Information Sources and Search Strategy

Multiple search strategies were employed. A systematic search with no publication date restriction was conducted in EMBASE (Ovid), MEDLINE (Ovid), CINAHL (Ebsco), PsycINFO (Ovid), and Scopus. Database searches took place on February 12th and 13th, 2024. The search was re‐run August 23rd, 2024, but we did not identify any additional studies. The search strategy was developed from the PEO framework [27] in collaboration with a research librarian. The search profile was first developed for EMBASE (Table 1) and subsequently adapted for each database [28]. Search profiles for MEDLINE, CINAHL, PsycINFO, and Scopus are available as supplemental material. Preliminary searches for gray literature were conducted in the National Cancer Institute's database and Google Scholar; however, no relevant references were identified. Furthermore, by searching in EMBASE we are also searching for gray literature and preprints in BioRxiv and medRxiv. The first author conducted the search.

**TABLE 1:** Search profile for EMBASE.^a^

Set	Search statement
#1	Immunotherapy/
#2	(immunotherap^a^ or immune therap^a^ or immunogenic therap^a^ or immunological therap^a^ or immunological treatment^a^ or immunomodula^a^ therap^a^).ti,ab,kf.
#3	Cancer immunotherapy/
#4	((cancer adj3 immunotherap^a^) or (tumor^a^ adj3 immunotherap^a^) or (tumour^a^ adj3 immunotherap^a^)).ti,ab,kf.
#5	Checkpoint inhibitor therapy/
#6	Immune checkpoint inhibitor/
#7	(check point blocking therap^a^ or check point inhibit^a^ therap^a^ or checkpoint blockade antibody therap^a^ or checkpoint blockade immune therap^a^ or checkpoint blockade immunotherap^a^ or checkpoint block^a^ therap^a^ or checkpoint block^a^ immune therap^a^ or checkpoint blocker therap^a^ or checkpoint blocking antibody therap^a^ or checkpoint blocking immunotherapy^a^ or checkpoint blocking therap^a^ or checkpoint immune therap^a^ or checkpoint immunotherap^a^ or checkpoint inhibit^a^ therap^a^ or checkpoint inhibit^a^ antibody therap^a^ or immune checkpoint block^a^ therap^a^ or immune checkpoint inhibit^a^ therap or immune checkpoint therap^a^ or immune‐checkpoint therap^a^ or immunocheckpoint therap^a^ or immunological checkpoint therap^a^ or inhibitor checkpoint therap^a^ or immune checkpoint inhibit^a^ therap^a^).ti,ab,kf.
#8	Cytotoxic T lymphocyte antigen 4/
#9	(cytotoxic T lymphocyte antigen 4 or antigen CD152 or CD152 antigen or CTLA 4 or ctla4 or cytotoxic T lymphocyte associated antigen 4 or CTLA‐4).ti,ab,kf.
#10	Cytotoxic T lymphocyte antigen 4 antibody/
#11	(cytotoxic T lymphocyte antigen 4 antibody or CD152 antibody or CTLA 4 antibody or CTLA4 antibody or anti‐CTLA‐4).ti,ab,kf.
#12	Programmed death 1 ligand 1/
#13	(programmed death 1 ligand 1 or antigen B7 H1 or antigen B7H1 or antigen CD274 or antigens, CD274 or B7H1 antigen or B7 H1 protein or B7 homolog 1 protein or B7H1 antigen or B7H1 protein or CD274 antigen^a^ or PDCD1 ligand 1 or PDCD1LG1 protein or programmed cell death 1 ligand 1 or programmed death 1 ligand 1 protein or programmed death ligand 1 or protein B7 H1 or protein B7H1 or protein PDCD1LG1 or PD‐L1 or anti‐PD‐L1 or programmed cell death ligand 1).ti,ab,kf.
#14	Programmed death 1 receptor/
#15	(programmed death 1 receptor or antigen CD279 or CD279 antigen or PD 1 protein or PDCD1 protein or programmed cell death 1 protein or programmed cell death 1 receptor or programmed cell death protein 1 or programmed death 1 protein or programmed death protein 1 or protein PD 1 or protein PDCD1 or protein programmed cell death 1 or protein programmed death 1 or PD‐1 or anti‐PD‐1 or programmed cell death protein 1 receptor).ti,ab,kf.
#16	Antineoplastic monoclonal antibody/
#17	antineoplastic^a^ monoclonal antibod.^a^ti,ab,kf.
#18	Immunological antineoplastic agent/
#19	(Immunological atineoplastic^a^ agent^a^ or antineoplastic^a^ agent,^a^ immunological or antineoplastic or immunosuppress^a^ agent^a^ or immunological anti cancer drug or immunological anti neoplastic agent^a^ or immunological anticancer agent^a^ or immunological anticancer drug or immunological anticarcinogen or immunological articarcinogenic agent^a^ or immunological antineoplastic agent^a^ or immunological antineoplastic drug or immunological antitumor agent^a^ or immunological antitumor drug or immunological antitumor agent or immunological antitumor drug or immunological cancer inhibit^a^ or immunological tumor inhibit^a^ or immunological tumour inhibit^a^).ti,ab,kf.
#20	Monoclonal antibody/
#21	(monoclonal antibod^a^ or antibod,^a^ monoclonal or antibod,^a^ monoclonal, humanized or antibod,^a^ monoclonal or clonal antibod^a^).ti,ab,kf.
#22	#1 or #2 or #3 or #4 or #5 or #6 or #7 or #8 or #9 or #10 or #11 or #12 or #13 or #14 or #15 or #16 or #17 or #18 or #19 or #20 or #21
#23	Malignant neoplasm/
#24	(cancer^a^ or carcinoma^a^ or malignant neoplas^a^ or malignant neoplas^a^ disease or malignant tumor^a^ or malignant tumour^a^ or neoplas^a^ malignan^a^ or oncologic^a^ malignan^a^ or tumor^a^ malignan^a^ or tumour^a^ malignan^a^).ti,ab,kf.
#25	Advanced cancer/
#26	(advanced cancer^a^ or cancer,^a^ advanced).ti,ab,kf.
#27	Disseminated cancer/
#28	(disseminated cancer^a^ or cancer,^a^ disseminated).ti,ab,kf.
#29	Early cancer/
#30	(early cancer^a^ or cancer,^a^ early or early carcinoma).ti,ab,kf.
#31	Solid malignant neoplasm/
#32	(solid malignant neoplasm or malignant neoplasm,^a^ solid or malignant solid tumor^a^ or malignant solid tumour^a^ or solid cancer^a^ or solid malignan^a^ or solid malignan^a^ neoplas^a^ or solid malignan^a^ tumor^a^ or solid malignan^a^ tumour^a^).ti,ab,kf.
#33	Solid tumor/
#34	(solid tumor^a^ or solid tumour^a^ or solid neoplas^a^).ti,ab,kf.
#35	Neoplasm/
#36	(neoplas^a^ or neoplastic disease or neoplastic entity or neoplastic mass or tumor^a^ or tumour^a^ or tumor^a^ entity or tumor^a^ mass or tumour^a^ entity or tumour^a^ mass).ti,ab,kf.
#37	#23 or #24 or #25 or #26 or #27 or #28 or #29 or #30 or #31 or #32 or #33 or #34 or #35 or #36
#38	Patient education/
#39	(patient education^a^ or education, patient or patient education as topic or patient medication knowledge or client education).ti,ab,kf.
#40	Health education/
#41	(health education or education, health or health fairs or health science^a^ education).ti,ab,kf.
#42	Medical information/
#43	(medical information or health communication or health information or information, medical).ti,ab,kf.
#44	Patient information/
#45	(patient information or self‐management education or information leaflet^a^ or health knowledge or client education or consumer^a^ health education).ti,ab,kf.
#46	Counseling/
#47	(counseling or counselling).ti,ab,kf.
#48	Patient counseling/
#49	(counseling, drug or counselling, drug or drug counseling or drug counselling).ti,ab,kf.
#50	#38 or #39 or #40 or #41 or #42 or #43 or #44 or #45 or #46 or #47 or #48 or #49
#51	#22 and #37 and #50

^a^ The search strategy was developed in collaboration with a research librarian, who recommended using search terms related to “immune checkpoint inhibitor” AND “cancer” AND “patient education”. Including search terms related to “self‐efficacy” and “self‐management” would increase the risk of missing important references.

### 2.4 Selection Process

The review's study selection process involved two steps: (a) screening titles and abstracts, and (b) screening full articles [29]. The first and last author independently screened titles and abstracts of all references using the screening and data extraction software Covidence [30]. References that failed to meet eligibility criteria or did not address the research question were excluded. Inconsistent screening results were resolved by discussion between the first and last author. The first and last authors then screened the full text of the included reports for relevance to the review question and eligibility criteria. Questions about study eligibility were resolved through discussion. The first author conducted author searches on authors of identified literature included in this review, and a backward citation search and forward citation tracking on included reports [31]. Furthermore, the “Find Similar”‐algorithm was applied in the searched databases.

### 2.5 Data Extraction and Analysis

The first author extracted the data into a predefined data extraction template in Covidence. Extracted data included author(s), year of publication, country of origin, study aim(s), outcomes, study design, setting(s), participant information, type of patient education provided, main findings, and results of the critical appraisal. Extracted data of the main findings were analyzed using thematic analysis [32]. The first, second, and last authors coded the data, identified patterns, and grouped the codes into themes. The thematic analysis is available as supplemental material.

### 2.6 Quality Appraisal

The Mixed Methods Appraisal Tool (MMAT) was used to critically appraise the quality of the included studies [33]. Exclusion based on the overall score is discouraged because integrative reviews are intended to gather and report all the evidence on a topic, regardless of the methodological quality [34]. Therefore, we did not exclude studies based on the results of the quality appraisal. The first author performed the critical appraisal and discussed the results with the last author.

## 3 Results

### 3.1 Search Results

**FIGURE 1:**
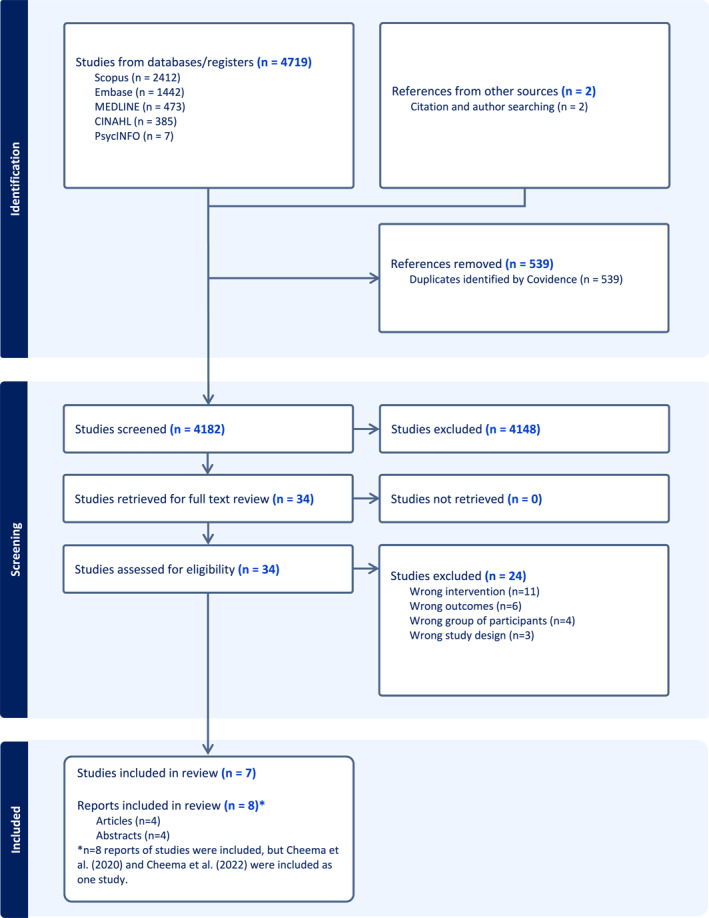
PRISMA Flow Diagram; *n* = 7 included studies.

After removing duplicates, 4182 references were screened for eligibility. Seven studies met the eligibility criteria and were included (see PRISMA Flow Diagram in Figure 1).

Included studies were conducted in the United States (*n* = 2) [35, 36], Canada (*n* = 2) [37, 38], Italy (*n* = 1) [39], Spain (*n* = 1) [40], and Germany (*n* = 1) [41]. Six studies included patients [35, 37, 38, 39, 40, 41], and one study included both patients and caregivers [36]. All the included studies provided quantitative data. The studies had various designs; one randomized controlled pilot trial [41], one cohort study [37], three pre‐post studies [35, 36, 38], and two observational studies [39, 40] (Table 2). We identified one study protocol [50] that met the inclusion criteria, but we decided to exclude as it is an ongoing study.

**TABLE 2:** Summary of eight included studies in a review of how patient education on immune checkpoint inhibitors affect patients with cancer and their family caregivers self‐efficacy and self‐management when dealing with immune‐related adverse events.

Authors, year of publication, journal, country	Aim	Design and outcomes	Patient education	Participants characteristics		Main results	Quality appraisal (“yes”/all checklist items)
Sauer et al. (2024) [41] *Cancer* Germany	To investigate the feasibility and acceptability and preliminary efficacy of SOFIA regarding health related quality of life, psychosocial symptoms, and medical data in routine clinical care	Randomized controlled pilot trial Primary outcomes: Feasibility by looking at recruitment rate, refusal and dropout rates, reasons for refusal and dropout, willingness to be randomized, utilization rate of SOFIA, and sample size estimation. Acceptability of SOFIA using Patient Feedback Form and a self‐developed questionnaire for physicians [42]. Health related quality of life using European Organization of Research and Treatment of Cancer Quality of Life of Cancer Patients questionnaire (EORTC QLQ‐C30) [43]. Secondary outcomes: Self‐reported symptoms of depression using Patient Health Questionnaire 0 (PHQ‐9; depression module) [44]. Anxiety levels using German General Anxiety Disorder Scale 7 [44]. Patients’ distress using the National Comprehensive Cancer Network distress thermometer (NCCN‐DT) [45]. Physician‐patient communication using the Physician‐Patient Interaction questionnaire (FAPI) [46]. Self‐efficacy using the Cancer Behavior Inventory‐Brief Version (CBI‐B) [47]. Patient treatment satisfaction was assessed with one item (How satisfied are you overall with the medical treatment so far) Rating: 1, not at all; 5, very much).	Intervention group: SOFIA App that consist of two components; SOFIA monitoring (ePROs that participants had to report twice a week) and SOFIA coaching (provide information about mental health, immunotherapy, nutrition, sports, social law, and contact information).	Number of included patients: *n* = 71 (dropouts: *n* = 21). Total number of patients: *n* = 50. Demographics at baseline on *n* = 71 patients. Intervention group (*n* = 34): age, mean = 59, 79, *n* = 20 men and *n* = 14 women, types of cancers: urological (*n* = 13), gastrointestinal (*n* = 9), hepatobiliary (*n* = 5), head and neck (*n* = 1), bronchial (*n* = 1), gynecological (*n* = 3), squamous cell (*n* = 2), treatment: PD‐1/PD‐L1 monotherapy (*n* = 11), PD‐1/CTLA‐4 combination therapy (*n* = 6), PD‐1/PD‐L1 + tyrosin kinase inhibitor (*n* = 10), PD‐1/PD‐L1 + chemotherapy (*n* = 7). Metastasis: *n* = 31. Palliative treatment: *n* = 34. Control group (*n* = 37): age, mean = 61, 65, *n* = 28 men and *n* = 9 women, types of cancers: urological (*n* = 16), gastrointestinal (*n* = 9), hepatobiliary (*n* = 5), head and neck (*n* = 3), bronchial (*n* = 3), gynecological (*n* = 0), squamous cell (*n* = 1), treatment: PD‐1/PD‐L1 monotherapy (*n* = 10), PD‐1/CTLA‐4 combination therapy (*n* = 4), PD‐1/PD‐L1 + tyrosin kinase inhibitor (*n* = 14), PD‐1/PD‐L1 + chemotherapy (*n* = 9). Metastasis: *n* = 32. Palliative treatment: *n* = 36.		SOFIA showed high feasibility and acceptance (retention rate of 85%). No difference regarding self‐efficacy from T0 to T1 (CBI‐B). Use of ePRO. More emergency room visits in the control group (*n* = 11) compared to the intervention group (*n* = 4) (*p* = 0.09). Patients in the intervention group showed significantly better HRQOL (*p* = 0.013) as well as less depression (*p* = 0.006) and distress (*p* = 0.006) compared to the control group.	6/7
Myers et al. (2023) [37] *Journal of Oncology Pharmacy Practice* Canada	To summarize the spectrum of patient care interventions performed by outpatient oncology pharmacists in those treated with immune checkpoint inhibitors (ICIs), and to determine if these interventions improve clinical outcomes focusing on immune‐related adverse events (irAE) education, irAE monitoring, irAE assessment and irAE management	Cohort study Clinical outcomes: The following clinical patient outcomes were collected: Total emergency department visits not resulting in admission to hospital. Hospitalization due to suspected irAEs. ICI treatment discontinuation due to irAEs. Total ICI cycles received. Completion of PD‐1/PD‐L1 inhibitors with a finite treatment course, if applicable. Completion of ipilimumab treatment **course**, if applicable.	Pharmacist intervention focused on irAE education, monitoring, assessment and management. After prescription to ICI, pharmacist educated patients and conducted a baseline assessment. Next, patients were provided contact information. Patients were scheduled for phone follow up prior to each cycle of ICI for the first 3 months of treatment. They compared with a matched control group, who did not receive intensive pharmacist intervention.	*n* = 143 patients in cohort group. Age, mean = 70.1, *n* = 85 men and *n* = 58 women, types of cancers: lung cancer (*n* = 96), malignant melanoma (*n* = 17), renal cell carcinoma (*n* = 17), urothelial carcinoma (*n* = 11), cutaneous squamous cell carcinoma (*n* = 1), missing data (*n* = 1), treatment: Atezolizumab (*n* = 11), Cemiplimab (*n* = 1), Durvalumab (*n* = 13), Ipilimumab (*n* = 1), Nivolumab (*n* = 60), Pembrolizumab (*n* = 48), Ipilimumab + Nivolumab (*n* = 9). *n* = 184 patients in control group (*n* = 92 pairs) (*n* = 92 received pharmacist intervention, and *n* = 92 did not receive pharmacist intervention). Age, mean, with (pharmacist intervention) = 69.2, age, mean, without pharmacist intervention = 68.8. Of them who received pharmacist intervention were *n* = 50 of them men and *n* = 42 women. Of them who did not receive pharmacist intervention were *n* = 48 men and *n* = 44 women. Types of cancer: lung cancer (*n* = 66), malignant melanoma (*n* = 15), renal cell carcinoma (*n* = 8), urothelial carcinoma (*n* = 3).		A higher odds of treatment discontinuation due to irAEs was identified in patients who did not receive dedicated pharmacist follow‐up (*p* = 0.022, OR = 5.5 (95% CI: 1.2–24.8).	6/7
Teixeira‐Poit et al. (2023) [35] *Seminars in Oncology Nursing* USA	To explore whether a refined patient education program for cancer ICI can (1) improve patient understanding about ICIs mechanisms of action, irAEs, and management of those and (2) reduce inappropriate emergency department utilization.	Pre‐post study Primary outcomes: Participants completed a survey before and after an ICI education session. Knowledge of cancer immunotherapy was operationalized using number and percentage of survey questions answered correctly. Healt literacy using Short Test of Functional Health Literacy in Adults (STOFHLA) [48]. Emergency department (ED) utilization was operationalized using the number of ED visits 6 months pre‐ and post‐intervention, and a qualitative review of the ED diagnoses and coded whether it would have been more appropriate for cancer ICI patients to present to the Symptom Management Clinic.	One‐on‐one 120 minutes education session from a clinical nurse educator before start of treatment, and includes an oral presentation, a video of ICI mechanisms of action, and written material on irAEs.	*n* = 38 patients. 52% of the patients were men, and 47% were women (missing data on 1%). 57% of the patients were retired. 97% of the patients had adequate health literacy (STOFHLA).		Cancer patients’ knowledge improved after the education (*m* = 83.1) compared to before the education session (*M* = 74.8). All patients after the education sessions recognized irAEs compared to 73% of patients before the education session. 97% of participants had adequate health literacy (STOFHLA). No difference in the number of emergency department visits, and no difference in the number of inappropriate emergency department visits.	5/7
Congiu & Webber (2021) [39] *Professioni Infermieristiche* Italy	To create and determine the acceptability of a readable tool to be shared with patients to provide information on irAEs and provide clinical guidance to prevent and manage symptoms.	Observational study Outcome: Acceptability of the readable tool using a questionnaire developed ad hoc.	A tool providing information on irAEs and providing clinical guidance to prevent and manage symptoms.		*n* = 35 patients. Age, median = 65 (SD = 10.7). Treated with: Pembrolizumab, Nivolumab, Atezolizumab and Durvalomab.	All patients find the educational tool useful and easy to understand for recognizing and managing irAEs. Patient education is of fundamental importance to ensure early identification of irAEs.	2/7
Cheema et al. (2020) [38] *Journal of Clinical Oncology* Canada	To study the impact of an immune‐oncology nursing baseline assessment, education and monitoring program on patient's irAE reporting and self‐efficacy of ICIs	Prospective pre‐post study Primary outcomes: Patient’s irAE reporting. Self‐efficacy using Cancer Behaviour Inventory – Brief Version (CBI‐B) [47].	Patients underwent a baseline nursing assessment and education class. The baseline assessment is used to document baseline symptoms to be able to establish a change in the patient’s clinical status when working up for irAEs, as well as to determine risks for irAEs. Patients identified at the assessment as high risk (risk of grade 3/4 irAE > 20%) had weekly nurse proactive calls.	*n* = 80 patients Age, median = 69, 70% men and 30% women, 81% had an ECOG performance status at 0–1 at baseline, 19% had elementary as highest education, 30% high school, 26% trade diploma and 21% post‐secondary, 41% had limited cancer health literacy. Types of cancer: NSCLC (55%), malignant melanoma (19%), renal cell carcinoma (9%), and other (17%), treatment: 70% PD‐1/PD‐L1 monotherapy.		A statistically improvement in the average CBI‐B scores found pre and post assessment/education (*p* < 0.001). 41% of participants had limited cancer health literacy (6‐item Cancer Health Literacy Test). Method of detection of irAEs was mainly by patient self‐reporting (62%), followed by proactive calls (27%). 3 patients had detection of an irAE with an emergency department visit.	3/7
Cheema et al. (2022) [49] *Current Oncology* Canada	To provide an in‐depth description of what was included in the development of patient education materials	Prospective pre‐post study Development article Outcomes: N/A (see Cheema et al. 2020).	Patient education includes the following: One‐on‐one session with nurse (includes baseline assessment with New Patient Immunotherapy Baseline Assessment and Teaching Checklist) High risk patients (risk of grade 3/4 irAE > 20%) receive proactive weekly calls. Symptoms are documented using CIOSK New Patient Baseline Symptom Tracker Sheet. A 6 minute teaching video Booklet “Your Guide to Cancer Immunotherapy” Patients receive a wallet card and a symptom diary Patients are provided with a letter for their general practitioner Drug‐specific information sheets are provided A patient‐facing poster of irAEs is in each patient exam room	N/A (see Cheema et al. 2020).		The patient education material led to improved patient comprehension of irAEs, the ability to act on symptoms (patient self‐efficacy), and low rates of emergency room visits for irAEs.	N/A
Serra et al. (2019) [40] *Journal of Thoracic Oncology* Spain	To describe the implementation of a nursing program for cancer patients treated with ICI by an ICI nurse specialist.	Observational study Outcome: Number of referrals to specialists	Before starting ICI, patients receive education that includes possible irAEs and the emergency workup that must be followed in case of occurence. Patients also receive an educational booklet.	*n* = 176 patients (hereof *n* = 100 patients with lung cancer)		The implementation of the ICI nursing program has contributed to a greater patient satisfaction. The implementation of the ICI nursing program has contributed to an improvement of early detection and management of irAEs in cancer patients receiving ICI treatment.	1/7
Herrmann et al. (2017) [36] *Journal of Thoracic Oncology* USA	A educational initiative was developed to determine if online education modules could improve knowledge about treatment decisions and side effect management in advanced non‐small cell lung cancer	Pre‐post study Outcome: Impact of knowledge using a pre‐/post‐activity survey.	Four educational activities available on a website dedicated to patient/caregiver learning. Each activity included demographic questions and a pre‐post‐activity question to measure impact on knowledge.	*n* = 8933 persons (either patients or caregivers) had participated in the education. 43% had lung cancer or were caregivers of a person with the disease, and 65% were women.		Online patient/caregiver‐focused education can be successful in improving familiarity with essential elements involved in treatment with ICI. There was an 8% increase in comprehending that irAEs should be reported to their cancer care team. There was a 16% increase in understanding the mechanism of action associated with use of ICI (*p* = 0.001). Targeted and focused digital education empowers, engages, and equips patient/caregiver with information needed for self‐care condition management.	3/7

The seven studies included a total of 9639 participants. The studies included patients with various cancer types: lung (*n* = 5) [36, 37, 38, 40, 41], melanoma (*n* = 2) [37, 38], renal cell (*n* = 2) [37, 38], squamous cell (*n* = 2) [37, 41], urological cancer (*n* = 2) [37, 41], hepatobiliary cancer (*n* = 1) [41], head and neck cancer (*n* = 1) [41], gynecological cancer (*n* = 1) [41] and gastrointestinal cancer (*n* = 1) [41]. Two studies did not specify the cancer diagnoses [35, 39], and four studies had missing data on some of the participants [36, 37, 38, 40]. Patients in the included studies received treatment with ICI as PD‐1/PD‐L1 monotherapy (*n* = 4) [37, 38, 39, 41], CTLA‐4/PD‐1 combination therapy (*n* = 3) [37, 38, 41], and PD‐1/PD‐L1 in combination with chemotherapy or other therapies (*n* = 2) [38, 41]. Data concerning types of ICI treatment is missing in *n* = 3 studies [35, 36, 40]. In two studies, ICI treatment was initiated due to metastatic disease [37, 41]. Data concerning the intend of ICI treatment is missing in *n* = 5 studies [35, 36, 38, 39, 40].

Notably, the studies reported as articles scored higher in the quality appraisal (mean score: 5.66 “yes”/all checklist items) [35, 37, 41] than the studies reported in abstracts (mean score: 2.25 “yes”/all checklist items) [36, 38, 39, 40] (Table 2) (quality appraisal using MMAT is available as supplemental material).

### 3.2 Synthesis and Reporting of Study Themes

**FIGURE 2:**
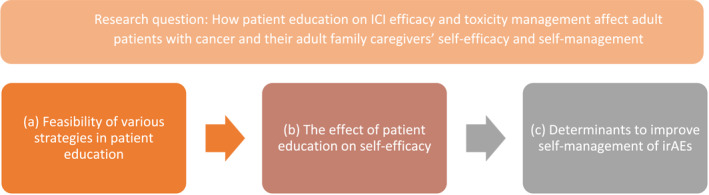
Three themes emerged; (a) Feasibility of various strategies in patient education, (b) The effect of patient education on self‐efficacy, and (c) Determinants to improve self‐management of irAEs. Patient education positively affects self‐efficacy, which in turn improves self‐management of irAEs.

Three themes emerged from the data: (a) Feasibility of various strategies in patient education, (b) The effect of patient education on self‐efficacy, and (c) Determinants to improve self‐management of irAEs (Figure 2).

#### 3.2.1 Feasibility of Various Strategies in Patient Education

Various strategies were identified for educating patients about irAEs. Of the seven included studies, one study examined patient education by the use of a smartphone application [41]; one study examined a pharmacist intervention including ICI and irAEs education prior to treatment initiation and proactive irAEs monitoring via telephone follow‐up prior to each cycle of ICI for the first three months [37]; one study tested online education modules [36]; one study examined a combined oral and written information and a video [35]; one study oral and written information [40]; one study only written information [39]; and one study as a baseline nursing assessment of baseline symptoms combining a one‐on‐one education session between the patient and ICI nurse and follow‐up phone calls [38].

Information leaflets providing information on irAEs and offering clinical guidance to prevent and manage symptoms can be useful for recognizing and managing irAEs if patients and caregivers actually read them [39]. In combination with oral education on ICIs it can contribute to greater patient satisfaction [40]. However, more innovative ways of educating patients with cancer and their caregivers about ICIs have also shown to be feasible. Online patient and caregiver‐focused education can be successful in improving familiarity with essential elements involved in treatment with ICIs, including irAEs [36]. Sauer et al. studied the feasibility of a smartphone application (the SOFIA‐App) with integrated ICI‐coaching and monitoring components. They found high feasibility and acceptance of the SOFIA‐App with a retention rate of 85% after three months [41]. Moreover, no patients refused participation following randomization, indicating the feasibility of the smartphone application [41]. These studies testing the feasibility of patient education offer hope for increased self‐efficacy and thereby better self‐management of irAEs.

#### 3.2.2 The Effect of Patient Education on Self‐Efficacy

Two of the included studies used the Cancer Behavior Inventory‐Brief Version (CBI‐B) [47] to evaluate patients' self‐efficacy [38, 41].

In general, patient education had a positive effect on patients' self‐efficacy. Cheema et al. found a statistically improvement in the average CBI‐B scores (*p* < 0.001) after a baseline nursing assessment of symptoms and education, and follow‐up phone calls [38], indicating an improved self‐efficacy and ability to act on irAEs. Similarly, pharmacist interventions including baseline education and follow‐up phone calls can empower the patient to play an active role in their cancer care, and improve detection of new symptoms or irAEs [37]. This aligns with Teixeira‐Poit et al. who found that patients' knowledge of treatment with ICIs improved after an education session compared to before the education session. Moreover, the patients were able to recognize irAEs after an education session [35]. Cheema et al. also found that a nursing assessment of symptoms and education program led to improved comprehension of irAEs [38].

Digital education can also empower, engage, and equip patients and their caregivers with valuable information needed for self‐care management of irAEs [36]. However, in a randomized controlled pilot study, Sauer et al. did not find any difference regarding self‐efficacy between the intervention group (the patients who used the SOFIA‐App) and the control group (the patients who received information and were provided with an emergency telephone number) [41].

Two studies assessed the patients' health literacy. Teixeira‐Poit et al. used the Short Test of Functional Health Literacy in Adults (STOFHLA) [51] to assess health literacy. They found that 97% of patients had adequate overall health literacy [35]. This is contrary to the findings of Cheema et al. who found that 41% of patients had limited cancer health literacy (using the 6‐item Cancer Health Literacy Test [52]).

#### 3.2.3 Determinants to Improve Self‐Management of irAEs

Patient education is vital to improve early detection and management of irAEs [39, 40]. Positive trends are especially apparent in the studies where patient education is combined with follow‐up phone calls or electronic Patient Reported Outcomes (ePRO). That the patients' reported outcomes are seen and acted upon by healthcare professionals may contribute to better health‐related quality of life as well as less depression and distress [41]. Myers et al. found higher odds of treatment discontinuation due to irAEs in patients who did not receive the dedicated pharmacist follow‐up [37]. Cheema et al. found that after a baseline nursing assessment of baseline symptoms and education, the method of irAEs detection was mainly by patient self‐reporting, which was the case in 62% of the reported irAEs, followed by proactive phone calls (27% of reported irAEs) [38]. Three patients had detection of an irAE during a visit to the emergency department [38]. This finding is similar to Sauer et al. who identified a trend toward more emergency department visits in the control group (*n* = 11) compared to the intervention group (*n* = 4). However, Teixeira‐Poit et al. found no differences in the number of emergency department visits before and after implementing ICI education in the form of oral information combined with a video of ICIs mechanisms of action and written material [35].

## 4 Discussion

To the best of our knowledge, this study is the only integrative review of evidence related to how patient education on ICI efficacy and toxicity management affects adult patients with cancer and their adult family caregivers' self‐efficacy and self‐management when dealing with immune‐related adverse events.

### 4.1 Search Results

We found few relevant studies, and they were all quantitative studies. All the included studies have been published within the last seven years, indicating that research within patient and caregiver education on ICI is in its infancy. The geographical diversity of the included studies underscores the global relevance of the research on how patient education on ICI affects patients with cancer and their family caregivers' self‐efficacy and self‐management in relation to irAEs. This geographical spread enhances the generalizability of the findings, although there is a predominance of Western countries.

The studies predominantly focused on patients with cancer, with only one study incorporating both patients and caregivers [36]. This indicates a gap in the research regarding the caregiver perspective. Patients manage their disease with daily assistance from family caregivers, and family caregivers' support may help empower and engage the patient to self‐manage the condition [53]. Therefore, educating family caregivers about ICI and irAEs is important.

The diversity on cancer types across the studies is notable, with lung cancer being the most frequent (included in *n* = 5 studies). This focus on lung cancer aligns with its high prevalence and the indication for the use of ICI on this large group of patients with cancer. Other major groups of cancer diagnoses, such as colorectal, breast, and prostate, have minimal use of ICI. The inclusion of a variety of cancer types broadens the understanding of patient experiences across different cancer diagnoses. However, the fact that two studies did not specify the cancer diagnosis and four studies had some missing data concerning cancer diagnosis is a limitation, as it restricts the ability to compare across specific cancer types.

### 4.2 Study Themes

In the following the three themes will be discussed: Feasibility of various strategies in patient education, The effect of patient education on self‐efficacy, and Determinants to improve self‐management of irAEs.

Various types of patient education (e.g., oral and/or written information) can be useful for recognizing and managing irAEs and contribute to a greater patient satisfaction. Innovative ways, such as online education or applications, are also feasible in improving familiarity with essential elements involved in treatment with ICI and irAEs. In general, patient education has a positive effect on patient's self‐efficacy, and empowers patients and their caregivers with valuable information needed for increased self‐management of irAEs.

The included studies present a diverse array of methods for educating patients and family caregivers about ICI and managing of irAEs. Several studies highlighted the effectiveness of combining oral and written information for patient education. The combination of educational strategies seems to increase patient satisfaction [40]. A study comparing written information on chemotherapy with audio‐visual education showed that audio‐visual education is more beneficial in reducing distress in patients with cancer [54]. Similarly, innovative methods are proven to be highly feasible educational tools to improve familiarity with key aspects of ICI treatment and irAEs [36, 41]. The positive effect of innovative methods in patient education is highlighted in a systematic review finding that the use of mHealth interventions can improve the patient's knowledge by the provision of self‐care recommendations, activation of the patient's role in the care process, improve the quality of life in patients with cancer, and contribute to patient satisfaction [55]. Looking for the future, virtual reality (VR) is emerging as an exciting avenue for patient education and can improve patient's knowledge of their illness and satisfaction with treatment [23, 50]. VR is already used in the cancer setting, however, most prevalent in radiotherapy [56].

Patient education interventions demonstrated a positive impact on self‐efficacy [38], which is in line with Myers et al. (2023) finding that patient education interventions empowered patients to communicate irAEs and manage their treatment. This aligns with a cross‐sectional study finding that patients with increased self‐efficacy had better chemotherapy self‐management [19], suggesting that structured educational programs can enhance patients' proactive involvement and confidence in managing irAEs.

Teixeira‐Poit et al. found that 97% of patients had adequate health literacy [35], which likely facilitated the effective uptake of educational content. On the contrary, Cheema et al. identified that 41% of patients had limited health literacy [38]. These contrasting findings underscore the importance of assessing and accommodating health literacy in patient education programs. Sørensen et al. also emphasized that having a low level of health literacy or no support from caregivers have a negative impact on patient safety regarding patient‐reported irAEs [57]. Similarly, another study found that patients with little social support or support network have poorer oncologic outcomes [58]. Therefore, patient education on ICI should include an examination of patients' health literacy and supportive network.

The importance of patient education on the early detection and management of irAEs in cancer treatment cannot be overstated. Structured patient education improves patient outcomes by enhancing their ability to detect and manage irAEs effectively [37, 38, 39, 40, 41]. In combination with follow‐up phone calls or ePROs, it can reduce treatment discontinuation [37], lead to fewer visits to the emergency department due to irAEs [37, 41], and increase patients' health‐related quality of life [41]. This is in line with Tolstrup et al., who emphasized the increase in patients' health‐related quality of life and cancer well‐being with the use of ePRO [59]. Furthermore, ePRO may enhance patients' awareness of irAEs and increase their feeling of involvement [60].

### 4.3 Implications

The findings of this integrative review highlight the critical role of patient education in managing irAEs for patients with cancer undergoing ICI treatment. Incorporating educational strategies combining written, oral, and digital resources to enhance patient and caregiver understanding of ICI are feasible to improve patient self‐efficacy and self‐management capabilities, leading to better clinical outcomes such as early detection and management of irAEs. The inclusion of follow‐up mechanisms, e.g., ePROs, can aid the early detection of irAEs, and improve overall patient satisfaction and quality of life. However, there is a lack of emphasis on educating the caregivers together with the patient, and future research should strive to include caregivers as they are of great importance to the patient during treatment. Furthermore, the promising results from innovative educational methods warrant further exploration. However, these future studies should focus on tailored educational material to accommodate varying levels of health literacy, ensuring that all patients and caregivers can benefit from these educational programs.

### 4.4 Limitations

All efforts were made to identify relevant studies, with two authors performing the reference screening. The study adhered to guidelines for integrative reviews, incorporating both primary studies and gray literature to provide complementary knowledge. A potential limitation was the selection of search terms, especially those related to patient education. Other search terms may have been relevant to include in the search, for example, information, and there is a risk that we have been missing relevant studies to include. However, including information as a search term could have increased the sensitivity of the database searches, resulting in a higher number of irrelevant studies. Furthermore, we did not include self‐efficacy and self‐management as search terms, as this would limit the number of hits too much and increase the risk of missing important studies. It is a limitation that four out of seven included studies are reported in the form of abstracts, limiting the provision of the full information on methodological considerations. In particular, information on sampling strategy and participant representativeness are not provided, which is reflected in the quality appraisal of the studies.

## 5 Conclusion

Limited evidence exists related to how patient education on ICI efficacy and toxicity management affects adult patients with cancer and their adult family caregivers' self‐efficacy and self‐management when dealing with immune‐related adverse events. Patient education is of fundamental importance to improve the early detection and management of irAEs. The findings indicate that while traditional methods of patient education (i.e., oral and/or written information) remain valuable, the integration of digital and innovative technologies holds significant promise to enhancing patient and caregiver understanding of ICI and irAEs. Furthermore, patient education combined with follow‐up (such as phone calls or ePRO) may contribute to better health‐related quality of life. In general, patient education has a positive effect on patients' self‐efficacy. However, health literacy impacts patients' ability to understand and manage their treatment and irAEs, emphasizing the need for personalized education approaches.

## Author Contributions

First author contributed in conceptualization, data curation, analysis, funding acquisition, investigation, methodology, project coordination, visualization, and writing the original draft. Second author contributed in conceptualization, analysis, funding acquisition, methodology, validation, and reviewing and editing the manuscript. Third author contributed in conceptualization, funding acquisition, methodology, and reviewing and editing the manuscript. Fourth author contributed in conceptualization and reviewing and editing the manuscript. Last author contributed in conceptualization, analysis, funding acquisition, methodology, supervision, validation, and reviewing and editing the manuscript.

## Conflicts of Interest

The authors declare no conflicts of interest.

## Registration and Protocol

Review protocol can be accessed from PROSPERO. Amendments to information provided in the protocol are as follows: change of title, the sequence of self‐efficacy and self‐management in the research question, first, second, and last author coded and gathered data into themes together, not using NVIVO14 for data analysis due to small number of references.

## Supplemental Material

Search profiles for MEDLINE, CINAHL, PsycINFO, and Scopus, a matrix depicting the data analysis using thematic analysis, and quality appraisals using MMAT is available online.

## Supporting information

Table S1

Table S2

Table S3

Table S4

Table S5

Table S6

## Data Availability

The data that support the findings of this study are available from the corresponding author upon reasonable request.
