# Supplementary material for: Patient and Caregiver Education to Support Self‐Efficacy and Self‐Management During Immunotherapy—An Integrative Review
Source: Psychooncology. 2025 Feb 26;34(3):e70100. doi: 10.1002/pon.70100 (PMC11865008; doi:10.1002/pon.70100)
Supplement: Supplementary file 2 — Table S2 [file PON-34-e70100-s002.docx]

### **Supplemental material 2 – Search profile for CINAHL**

| **Supplemental material 2.** Search profile for CINAHL | |
| --- | --- |
| **Set** | **Search Statement** |
| #1 | Immunotherapy/ |
| #2 | (immunotherap* or immune therap* or immunogenic therap* or immunological therap* or immunological treatment* or immunomodula* therap*) |
| #3 | ((cancer N2 immunotherap*) or (tumor* N2 immunotherap*) or (tumour* N2 immunotherap*)) |
| #4 | Immune Checkpoint Inhibitors/ |
| #5 | (check point blocking therap* or check point inhibit* therap* or checkpoint blockade antibody therap* or checkpoint blockade immune therap* or checkpoint blockade immunotherap* or checkpoint block* therap* or checkpoint block* immune therap* or checkpoint blocker therap* or checkpoint blocking antibody therap* or checkpoint blocking immunotherapy* or checkpoint blocking therap* or checkpoint immune therap* or checkpoint immunotherap* or checkpoint inhibit* therap* or checkpoint inhibit* antibody therap* or immune checkpoint block* therap* or immune checkpoint inhibit* therap or immune checkpoint therap* or immune-checkpoint therap* or immunocheckpoint therap* or immunological checkpoint therap* or inhibitor checkpoint therap* or immune checkpoint inhibit* therap*) |
| #6 | (cytotoxic T lymphocyte antigen 4 or antigen CD152 or CD152 antigen or CTLA 4 or ctla4 or cytotoxic T lymphocyte associated antigen 4 or CTLA-4) |
| #7 | (cytotoxic T lymphocyte antigen 4 antibody or CD152 antibody or CTLA 4 antibody or CTLA4 antibody or anti-CTLA-4) |
| #8 | Programmed Cell Death Ligand 1/ |
| #9 | (programmed death 1 ligand 1 or antigen B7 H1 or antigen B7H1 or antigen CD274 or antigens, CD274 or B7H1 antigen or B7 H1 protein or B7 homolog 1 protein or B7H1 antigen or B7H1 protein or CD274 antigen* or PDCD1 ligand 1 or PDCD1LG1 protein or programmed cell death 1 ligand 1 or programmed death 1 ligand 1 protein or programmed death ligand 1 or protein B7 H1 or protein B7H1 or protein PDCD1LG1 or PD-L1 or anti-PD-L1 or programmed cell death ligand 1) |
| #10 | Programmed Cell Death Protein 1 Receptor/ |
| #11 | (programmed death 1 receptor or antigen CD279 or CD279 antigen or PD 1 protein or PDCD1 protein or programmed cell death 1 protein or programmed cell death 1 receptor or programmed cell death protein 1 or programmed death 1 protein or programmed death protein 1 or protein PD 1 or protein PDCD1 or protein programmed cell death 1 or protein programmed death 1 or PD-1 or anti-PD-1 or programmed cell death protein 1 receptor) |
| #16 | antineoplastic monoclonal antibody/ |
| #12 | (antineoplastic* monoclonal antibod*) |
| #13 | (immunological atineoplastic* agent* or antineoplastic* agent*, immunological or antineoplastic and immunosuppress* agent* or immunological anti cancer drug or immunological anti neoplastic agent* or immunological anticancer agent* or immunological anticancer drug or immunological anticarcinogen or immunological articarcinogenic agent* or immunological antineoplastic agent* or immunological antineoplastic drug or immunological antitumor agent* or immunological antitumor drug or immunological antitumour agent or immunological antitumour drug or immunological cancer inhibit* or immunological tumor inhibit* or immunological tumour inhibit*) |
| #14 | Antibodies, Monoclonal/ |
| #15 | (monoclonal antibod* or antibod*, monoclonal or antibod*, monoclonal, humanized or antibod*,monoclonal or clonal antibod*) |
| #16 | #1 OR #2 OR #3 OR #4 OR #5 OR #6 OR #7 OR #8 OR #9 OR #10 OR #11 OR #12 OR #13 OR #14 OR #15 |
| #17 | (cancer* or carcinoma* or malignant neoplas* or malignant neoplas* disease or malignant tumor* or malignant tumour* or neoplas* malignan* or oncologic* malignan* or tumor* malignan* or tumour* malignan*) |
| #18 | (advanced cancer* or cancer*, advanced) |
| #19 | (disseminated cancer* or cancer*, disseminated) |
| #20 | (early cancer* or cancer*, early or early carcinoma).ti,ab,kf. |
| #21 | (solid malignant neoplasm or malignant neoplasm*, solid or malignant solid tumor* or malignant solid tumour* or solid cancer* or solid malignan* or solid malignan* neoplas* or solid malignan* tumor* or solid malignan* tumour*) |
| #22 | (solid tumor* or solid tumour* or solid neoplas*) |
| #23 | Neoplasms/ |
| #24 | (neoplas* or neoplastic disease or neoplastic entity or neoplastic mass or tumor* or tumour* or tumor* entity or tumor* mass or tumour* entity or tumour* mass) |
| #25 | #17 OR #18 OR #19 OR #20 OR #21 OR #22 OR #23 OR #24 |
| #26 | Patient Education/ |
| #27 | (patient education* or education, patient or patient education as topic or patient medication knowledge or client education or pretraining) |
| #28 | Health Education/ |
| #29 | (health education or education, health or health fairs or health science* education) |
| #30 | (medical information or health communication or health information or information, medical) |
| #31 | (patient information or self-management education or information leaflet* or client education or consumer* health education) |
| #32 | Counseling/ |
| #33 | (counseling or counselling) |
| #34 | (counseling, drug or counselling, drug or drug counseling or drug counselling) |
| #35 | Health Knowledge/ |
| #36 | (health knowledge) |
| #37 | #26 OR #27 OR #28 OR #29 OR #30 OR #31 OR #32 OR #33 OR #34 OR #35 OR #36 |
| #38 | #16 AND #25 AND #37 |
