# Supplementary material for: Patient and Caregiver Education to Support Self‐Efficacy and Self‐Management During Immunotherapy—An Integrative Review
Source: Psychooncology. 2025 Feb 26;34(3):e70100. doi: 10.1002/pon.70100 (PMC11865008; doi:10.1002/pon.70100)
Supplement: Supplementary file 4 — Table S4 [file PON-34-e70100-s004.docx]

### **Supplemental material 4 – Search profile for Scopus**

| **Supplemental material 4.** Search profile for Scopus* | |
| --- | --- |
| **Set** | **Search Statement** |
| #1 | (immunotherap* or (immune W/0 therap*) or (immunogenic W/0 therap*) or (immunological W/0 therap*) or (immunological W/0 treatment*) or (immunomodula* W/0 therap*)) |
| #2 | ((cancer W/2 immunotherap*) or (tumor* W/2 immunotherap*) or (tumour* W/2 immunotherap*)) |
| #3 | ((check W/0 point W/0 blocking W/0 therap*) or (check W/0 point W/0 inhibit* W/0 therap*) or (checkpoint W/0 blockade W/0 antibody W/0 therap*) or (checkpoint W/0 blockade W/0 immune W/0 therap*) or (checkpoint W/0 blockade W/0 immunotherap*) or (checkpoint W/0 block* W/0 therap*) or (checkpoint W/0 block* W/0 immune W/0 therap*) or (checkpoint W/0 blocker W/0 therap*) or (checkpoint W/0 blocking W/0 antibody W/0 therap*) or (checkpoint W/0 blocking W/0 immunotherapy*) or (checkpoint W/0 blocking W/0 therap*) or (checkpoint W/0 immune W/0 therap*) or (checkpoint W/0 immunotherap*) or (checkpoint W/0 inhibit* W/0 therap*) or (checkpoint W/0 inhibit* W/0 antibody W/0 therap*) or (immune W/0 checkpoint W/0 block* W/0 therap*) or (immune W/0 checkpoint W/0 inhibit* W/0 therap) or (immune W/0 checkpoint W/0 therap*) or (immune-checkpoint W/0 therap*) or (immunocheckpoint W/0 therap*) or (immunological W/0 checkpoint W/0 therap*) or (inhibitor W/0 checkpoint W/0 therap*) or (immune W/0 checkpoint W/0 inhibit* W/0 therap*)) |
| #4 | ((cytotoxic W/0 T W/0 lymphocyte W/0 antigen W/0 4) or (antigen W/0 CD152) or (CD152 W/0 antigen) or (CTLA W/0 4) or ctla4 or (cytotoxic W/0 T W/0 lymphocyte W/0 associated W/0 antigen W/0 4) or CTLA-4) |
| #5 | ((cytotoxic W/0 T W/0 lymphocyte W/0 antigen W/0 4 W/0 antibody) or (CD152 W/0 antibody) or (CTLA W/0 4 W/0 antibody) or (CTLA4 W/0 antibody) or anti-CTLA-4) |
| #6 | ((programmed W/0 death W/0 1 W/0 ligand W/0 1) or (antigen W/0 B7 W/0 H1) or (antigen W/0 B7H1) or (antigen W/0 CD274) or (antigens, W/0 CD274) or (B7H1 W/0 antigen) or (B7 W/0 H1 W/0 protein) or (B7 W/0 homolog W/0 1 W/0 protein) or (B7H1 W/0 antigen) or (B7H1 W/0 protein) or (CD274 W/0 antigen*) or (PDCD1 W/0 ligand W/0 1) or (PDCD1LG1 W/0 protein) or (programmed W/0 cell W/0 death W/0 1 W/0 ligand W/0 1) or (programmed W/0 death W/0 1 W/0 ligand W/0 1 W/0 protein) or (programmed W/0 death W/0 ligand W/0 1) or (protein W/0 B7 W/0 H1) or (protein W/0 B7H1) or (protein W/0 PDCD1LG1) or PD-L1 or anti-PD-L1 or (programmed W/0 cell W/0 death W/0 ligand W/0 1)) |
| #7 | ((programmed W/0 death W/0 1 W/0 receptor) or (antigen W/0 CD279) or (CD279 W/0 antigen) or (PD W/0 1 W/0 protein) or (PDCD1 W/0 protein) or (programmed W/0 cell W/0 death W/0 1 protein) or (programmed W/0 cell W/0 death W/0 1 W/0 receptor) or (programmed W/0 cell W/0 death W/0 protein W/0 1) or (programmed W/0 death W/0 1 W/0 protein) or (programmed W/0 death W/0 protein W/0 1) or (protein W/0 PD W/0 1) or (protein W/0 PDCD1) or (protein W/0 programmed W/0 cell W/0 death W/0 1) or (protein W/0 programmed W/0 death W/0 1) or PD-1 or anti-PD-1 or (programmed W/0 cell W/0 death W/0 protein W/0 1 W/0 receptor)) |
| #8 | (antineoplastic* W/0 monoclonal W/0 antibod*) |
| #9 | ((immunological W/0 atineoplastic* W/0 agent*) or (antineoplastic* W/0 agent*, W/2 immunological) or (antineoplastic and immunosuppress* W/0 agent*) or (immunological W/0 anti W/0 cancer W/0 drug) or (immunological W/0 anti W/0 neoplastic W/0 agent*) or (immunological W/0 anticancer W/0 agent*) or (immunological W/0 anticancer W/0 drug) or (immunological W/0 anticarcinogen) or (immunological W/0 articarcinogenic W/0 agent*) or (immunological W/0 antineoplastic W/0 agent*) or (immunological W/0 antineoplastic W/0 drug) or (immunological W/0 antitumor W/0 agent*) or (immunological W/0 antitumor W/0 drug) or (immunological W/0 antitumour W/0 agent) or (immunological W/0 antitumour W/0 drug) or (immunological W/0 cancer W/0 inhibit*) or (immunological W/0 tumor W/0 inhibit*) or (immunological W/0 tumour W/0 inhibit*)) |
| #10 | ((monoclonal W/0 antibod*) or (antibod*, W/2 monoclonal) or (antibod*, W/2 monoclonal, W/2 humanized) or (antibod*, W/2 monoclonal) or (clonal W/2 antibod*)) |
| #11 | #1 OR #2 OR #3 OR #4 OR #5 OR #6 OR #7 OR #8 OR #9 OR #10 |
| #12 | (cancer* or carcinoma* or (malignant W/0 neoplas*) or (malignant W/0 neoplas* W/0 disease) or (malignant W/0 tumor*) or (malignant W/0 tumour*) or (neoplas* W/0 malignan*) or (oncologic* W/0 malignan*) or (tumor* W/0 malignan*) or (tumour* W/0 malignan*)) |
| #13 | ((advanced W/0 cancer*) or (cancer*, W/0 advanced)) |
| #14 | ((disseminated W/0 cancer*) or (cancer*, W/0 disseminated)) |
| #15 | ((early W/0 cancer*) or (cancer*, W/0 early) or (early W/0 carcinoma)) |
| #16 | ((early W/0 cancer*) or (cancer*, W/0 early) or (early W/0 carcinoma)) |
| #17 | ((solid W/0 malignant W/0 neoplasm) or (malignant W/0 neoplasm*, W/2 solid) or (malignant W/0 solid W/0 tumor*) or (malignant W/0 solid W/0 tumour*) or (solid W/0 cancer*) or (solid W/0 malignan*) or (solid W/0 malignan* W/0 neoplas*) or (solid W/0 malignan* W/0 tumor*) or (solid W/0 malignan* W/0 tumour*)) |
| #18 | ((solid W/0 tumor*) or (solid W/0 tumour*) or (solid W/0 neoplas*)) |
| #19 | (neoplas* or (neoplastic W/0 disease) or (neoplastic W/0 entity) or (neoplastic W/0 mass) or tumor* or tumour* or (tumor* W/0 entity) or (tumor* W/0 mass) or (tumour* W/0 entity) or (tumour* W/0 mass)) |
| #20 | #12 OR #13 OR #14 OR #15 OR #16 OR #17 OR #18 OR #19 |
| #21 | ((patient W/0 education*) or (education, W/2 patient) or (patient W/0 education W/0 as W/0 topic) or (patient W/0 medication W/0 knowledge) or (client W/0 education) or pretraining) |
| #22 | ((health W/0 education) or (education, W/2 health) or (health W/0 fairs) or (health W/0 science* W/0 education)) |
| #23 | ((medical W/0 information) or (health W/0 communication) or (health W/0 information) or (information, W/0 medical)) |
| #24 | ((patient W/0 information) or (self-management W/0 education) or (information W/0 leaflet*)) |
| #25 | ((counseling) or (counselling)) |
| #26 | ((counseling, W/2 drug) or (counselling, W/2 drug) or (drug W/0 counseling) or (drug W/0 counselling)) |
| #27 | ((health W/0 knowledge) or (client W/0 education) or (consumer* W/0 health W/0 education)) |
| #28 | #21 OR #22 OR #23 OR #24 OR #25 OR #26 OR #27 |
| #29 | #11 AND #20 AND #28 |

*Searching in Scopus is different from searching in the other databases. To increase readability, the search profile for Scopus is set up similarly to the search profile for the other databases.
