# Supplementary material for: Patient and Caregiver Education to Support Self‐Efficacy and Self‐Management During Immunotherapy—An Integrative Review
Source: Psychooncology. 2025 Feb 26;34(3):e70100. doi: 10.1002/pon.70100 (PMC11865008; doi:10.1002/pon.70100)
Supplement: Supplementary file 5 — Table S5 [file PON-34-e70100-s006.docx]

### **Supplemental material 5 – Thematic analysis**

| Meaning unit | Code | Category | Theme |
| --- | --- | --- | --- |
| SOFIA showed high feasibility and acceptance in patients in the intervention group. n=4 dropouts in the intervention group (n=2 patients died). Retention rate of 85% (41) | Feasibility and acceptance of app | Feasibility and acceptance of patient education material | Feasibiliy of various innovative strategies in patient education |
| CBI-B  No difference regarding self-efficacy from T0 to T1 (41) | Changes or no changes in self-efficacy | Self-efficacy and impact on irAEs management | The effect of patient education on self-efficacy |
| Using ePRO (41) | Patient Reported Outcome to help identify irAEs | Follow-up to identify irAEs | Determinants to improve self-management of irAEs |
| A trend toward more emergency room visits in the control group (n=11) compared to the intervention group (n=4) (*p*=.09).  No statistically significant differences in regard to rate and severity of irAEs and inpatient visits (41) | Differences in the use of emergency departments | Identification and management of irAEs | Determinants to improve self-management of irAEs |
| Patients in the intervention group showed significantly better HRQOL (*p*=.013) as well as less depression (*p*=.006) and distress (*p*=.006) compared to the control group (41) | App may lead to better outcomes | Identification and management of irAEs | Determinants to improve self-management of irAEs |
| The baseline and ongoing education provided during in-person and telephone follow-ups can indirectly improve communication of new symptoms by empowering the patient to play an active role in their own care (37) | Follow-up phone calls to help identify irAEs | Follow-up to identify irAEs | The effect of patient education on self-efficacy |
| A higher odds of treatment discontinuation due to irAEs was identified in patients who did not receive dedicated pharmacist follow-up (37) | Treatment discontinuation without follow-up | Follow-up to identify irAEs | Determinants to improve self-management of irAEs |
| STOFHLA (Short Test of Functional Health Literacy in Adults)  97% of participants had adequate health literacy (35) | Health literacy | Health literacy to increase self-efficacy | The effect of patient education on self-efficacy |
| No difference in the number of emergency department visits before the education session and after the education session.  Furthermore, no difference in the number of inappropriate emergency department visits (35) | Differences in the use of emergency departments | Identification and management of irAEs | Determinants to improve self-management of irAEs |
| All patients find the educational tool useful and easy to understand for recognizing and managing irAEs (39) | Feasibility and acceptance of written information | Feasibility and acceptance of patient education material | Feasibility of various innovative strategies in patient education |
| Patient education is of fundamental importance to ensure early identification of irAEs (39) | Patient education to ensure early identification of irAEs | Identification and management of irAEs | Determinants to improve self-management of irAEs |
| A statistically improvement in the average CBI-B scores found pre and post assessment/education (*p* <0.001) and this improvement was maintained over time at follow-up visits (38) | Improvement in self-efficacy | Self-efficacy and impact on irAEs management | The effect of patient education on self-efficacy |
| 41% of participants had limited cancer health literacy (CHLT6) (38) | Health literacy | Health literacy to increase self-efficacy | The effect of patient education on self-efficacy |
| Method of detection was mainly by patient self-reporting (62%), followed by proactice calls (27%). 3 patients had detection of an irAE with an emergency sdepartment visit (38) | Patient education to ensure early identification of irAEs | Identification and management of irAEs | Determinants to improve self-management of irAEs |
| The implementation of the ICI nursing program has contributed to a greater patient satisfaction (40) | Feasibility and acceptance of ICI nursing program | Feasibility and acceptance of patient education material | Feasibility of various innovative strategies in patient education |
| The implementation of the ICI nursing program has contributed to an improvement of early detection and management of irAEs in cancer patients receiving immunotherapy (40) | Patient education to ensure early identification of irAEs | Identification and management of irAEs | Determinants to improve self-management of irAEs |
| Online patient/caregiver-focused education can be successful in improving familiarity with essential elements involved in treatment with ICI (36) | Feasibility and acceptance of online education | Feasibility and acceptance of patient education material | Feasibility of various innovative strategies in patient education |
| Targeted and focused digital education empowers, engages and equips patient/caregiver with information needed for self-care condition management (36) | Online education empowers patients and caregivers | Self-efficacy and impact on irAEs management | The effect of patient education on self-efficacy |
