# Supplementary material for: Patient and Caregiver Education to Support Self‐Efficacy and Self‐Management During Immunotherapy—An Integrative Review
Source: Psychooncology. 2025 Feb 26;34(3):e70100. doi: 10.1002/pon.70100 (PMC11865008; doi:10.1002/pon.70100)
Supplement: Supplementary file 6 — Table S6 [file PON-34-e70100-s003.docx]

### **Supplemental material 6 – Quality appraisal using MMAT**

| **Sauer et al. 2024** | **Electronic health intervention to manage symptoms of immunotherapy in patients with cancer (SOFIA): Results from a randomized controlled pilot trial** | | | | |
| --- | --- | --- | --- | --- | --- |
| **Category of study designs** | **Methodological quality criteria** | **Responses** | | | |
|  |  | Yes | No | Can’t tell | Comments |
| Screening questions (for all types) | S1. Are there clear research questions? | X |  |  | Clear aim (no RQ) |
|  | S2. Do the collected data allow to address the research questions? | X |  |  |  |
| 2. Quantitative randomized controlled trials | 2.1. Is randomization appropriately performed? | X |  |  |  |
|  | 2.2. Are the groups comparable at baseline? | X |  |  | CG had slightly higher age, and more men allocated than the IG, but overall balanced baseline characteristics |
|  | 2.3. Are there complete outcome data? | X |  |  |  |
|  | 2.4. Are outcome assessors blinded to the intervention provided? |  | X |  |  |
|  | 2.5. Did the participants adhere to the assigned intervention? | X |  |  |  |

| **Myers et al. 2023** | **Evaluation and clinical impact of a pharmacist-led, interdisciplinary service focusing on education, monitoring and toxicity management of immune checkpoint inhibitors** | | | | |
| --- | --- | --- | --- | --- | --- |
| **Category of study designs** | **Methodological quality criteria** | **Responses** | | | |
|  |  | Yes | No | Can’t tell | Comments |
| Screening questions (for all types) | S1. Are there clear research questions? | X |  |  | Clear aim (no RQ) |
|  | S2. Do the collected data allow to address the research questions? | X |  |  |  |
| 3. Quantitative non-randomized | 3.1. Are the participants representative of the target population? | X |  |  |  |
|  | 3.2. Are measurements appropriate regarding both the outcome and intervention (or exposure)? | X |  |  |  |
|  | 3.3. Are there complete outcome data? | X |  |  |  |
|  | 3.4. Are the confounders accounted for in the design and analysis? |  |  | X |  |
|  | 3.5. During the study period, is the intervention administered (or exposure occurred) as intended? | X |  |  |  |

| **Teixeira-Poit et al. 2023** | **Efficacy of a Patient Education Session for Cancer Patients Receiving Immunotherapy** | | | | |
| --- | --- | --- | --- | --- | --- |
| **Category of study designs** | **Methodological quality criteria** | **Responses** | | | |
|  |  | Yes | No | Can’t tell | Comments |
| Screening questions (for all types) | S1. Are there clear research questions? | X |  |  |  |
|  | S2. Do the collected data allow to address the research questions? | X |  |  |  |
| 3. Quantitative non-randomized | 3.1. Are the participants representative of the target population? |  |  | X |  |
|  | 3.2. Are measurements appropriate regarding both the outcome and intervention (or exposure)? | X |  |  |  |
|  | 3.3. Are there complete outcome data? | X |  |  |  |
|  | 3.4. Are the confounders accounted for in the design and analysis? |  |  | X |  |
|  | 3.5. During the study period, is the intervention administered (or exposure occurred) as intended? | X |  |  |  |

| **Serra et al. 2019** | **Implementation of a Nursing Program for Cancer Patients Treated with Immunotherapy by an Immunotherapy Nurse Specialist** | | | | |
| --- | --- | --- | --- | --- | --- |
| **Category of study designs** | **Methodological quality criteria** | **Responses** | | | |
|  |  | Yes | No | Can’t tell | Comments |
| Screening questions (for all types) | S1. Are there clear research questions? | X |  |  | Clear aim (no RQ) |
|  | S2. Do the collected data allow to address the research questions? |  |  | X |  |
| 4. Quantitative descriptive | 4.1. Is the sampling strategy relevant to address the research question? |  |  | X |  |
|  | 4.2. Is the sample representative of the target population? |  |  | X |  |
|  | 4.3. Are the measurements appropriate? |  |  | X |  |
|  | 4.4. Is the risk of nonresponsive bias low? |  |  | X |  |
|  | 4.5. Is the statistical analysis appropriate to answer the research question? |  |  | X |  |

| **Congiu, M. & Webber, T.B. 2021** | **Educating the cancer patient about the immunotherapy toxicity: a descriptive observational study** | | | | |
| --- | --- | --- | --- | --- | --- |
| **Category of study designs** | **Methodological quality criteria** | **Responses** | | | |
|  |  | Yes | No | Can’t tell | Comments |
| Screening questions (for all types) | S1. Are there clear research questions? | X |  |  | Clear aim (no RQ) |
|  | S2. Do the collected data allow to address the research questions? | X |  |  |  |
| 4. Quantitative descriptive | 4.1. Is the sampling strategy relevant to address the research question? |  |  | X |  |
|  | 4.2. Is the sample representative of the target population? |  |  | X |  |
|  | 4.3. Are the measurements appropriate? |  |  | X |  |
|  | 4.4. Is the risk of nonresponsive bias low? |  |  | X |  |
|  | 4.5. Is the statistical analysis appropriate to answer the research question? |  |  | X |  |

| **Herrmann et al. 2017** | **Online Patient Education in Advanced Lung Cancer: Effect on Patient/Caregiver Knowledge** | | | | |
| --- | --- | --- | --- | --- | --- |
| **Category of study designs** | **Methodological quality criteria** | **Responses** | | | |
|  |  | Yes | No | Can’t tell | Comments |
| Screening questions (for all types) | S1. Are there clear research questions? | X |  |  | Clear aim (no RQ) |
|  | S2. Do the collected data allow to address the research questions? | X |  |  |  |
| 4. Quantitative descriptive | 4.1. Is the sampling strategy relevant to address the research question? |  |  | X |  |
|  | 4.2. Is the sample representative of the target population? |  |  | X |  |
|  | 4.3. Are the measurements appropriate? | X |  |  |  |
|  | 4.4. Is the risk of nonresponsive bias low? |  |  | X |  |
|  | 4.5. Is the statistical analysis appropriate to answer the research question? |  |  | X |  |

| **Cheema et al. 2020** | **Impact of an immune-oncology (IO) education/monitoring program on patient’s self-efficacy and adverse event reporting from immune checkpoint inhibitors (ICIs)** | | | | |
| --- | --- | --- | --- | --- | --- |
| **Category of study designs** | **Methodological quality criteria** | **Responses** | | | |
|  |  | Yes | No | Can’t tell | Comments |
| Screening questions (for all types) | S1. Are there clear research questions? | X |  |  | Aim (no RQ) |
|  | S2. Do the collected data allow to address the research questions? | X |  |  |  |
| 3. Quantitative non-randomized | 3.1. Are the participants representative of the target population? |  |  | X |  |
|  | 3.2. Are measurements appropriate regarding both the outcome and intervention (or exposure)? | X |  |  |  |
|  | 3.3. Are there complete outcome data? |  |  | X |  |
|  | 3.4. Are the confounders accounted for in the design and analysis? |  |  | X |  |
|  | 3.5. During the study period, is the intervention administered (or exposure occurred) as intended? |  |  | X |  |
